# Self-Reports in the Field Using Smartwatches: An Open-Source Firmware Solution

**DOI:** 10.3390/s22051980

**Published:** 2022-03-03

**Authors:** Selina Volsa, Bernad Batinic, Stefan Stieger

**Affiliations:** 1 Department of Psychology and Psychodynamics, Karl Landsteiner University of Health Sciences, 3500 Krems an der Donau, Austria; stefan.stieger@kl.ac.at; 2 Department of Work, Organizational and Media Psychology, Johannes Kepler University, 4040 Linz, Austria; bernad.batinic@jku.at

**Keywords:** experience sampling, ecological momentary assessment, smartwatch, wearable

## Abstract

In situ self-reports are a useful tool in the social sciences to supplement laboratory experiments. Smartwatches are a promising form factor to realize these methods. However, to date, no user-friendly, general-purpose solution has been available. This article therefore presents a newly developed, free and open-source firmware that facilitates the Experience Sampling Method and other self-report methods on a commercially-available, programmable smartwatch based on the ESP32 microcontroller. In a small-scale pilot study comparing this smartwatch and firmware to an equivalent design on smartphones, participants using the smartwatch showed increased compliance. The presented project demonstrates a useful tool for complementary tools like smartphones for self-reports.

## 1. Introduction

Compared to laboratory studies, field research offers high ecological validity, i.e., high generalizability of results in everyday life situations. This is important in sciences like psychology, sociology, and other social sciences that make claims about real-life experience and behavior. In situ self-reports are a valuable tool for the realization of field research but can be burdensome for participants. Smartwatches are a promising form factor to reduce this burden, as they are relatively unobtrusive. However, no easy-to-use general purpose software solution for realizing studies with these methods has been available so far. Thus, our intent is to present such a solution. 

### 1.1. The Experience Sampling Method

Historically a lot of research in the social sciences was conducted in the laboratory. However, laboratory studies often suffer from low ecological validity, as the laboratory setting is different from real life experience by design. Thus, methods in the field have been developed to complement laboratory methods. One such method is the Experience Sampling Method (ESM) [1], also known as Ambulatory Assessment (AA) or Ecological Momentary Assessment (EMA) [2,3].

The ESM is characterized by repeated measurements in a participant’s everyday life. These measurements take place over an extended period, from days to multiple weeks. This means that it can assess time courses with minimal bias in a setting the participants are familiar with, thus providing high ecological validity. Nonetheless, measuring often and in a way that is likely to interrupt participants in their everyday life can introduce a burden on participants, in turn decreasing their compliance to the study protocol. Therefore, reducing this burden is an important step to ensure high compliance and data quality.

Various forms of scheduling are common within the ESM [4], placing different demands on participants. In its simplest form, participants fill out questionnaires about their current situation when they are notified (i.e., signal-based), either at specific times or at pseudo-randomized times (i.e., randomized times within a predefined time frame). Participants might also be notified to similarly fill out questionnaires about a time interval prior to the notification (i.e., interval-based). Lastly, participants might be asked to fill out questionnaires whenever a specific event occurs within their everyday life (i.e., event-based, which participants detect on their own, meaning there is no notification involved). 

### 1.2. Smartwatches vs. Smartphones

To conduct an ESM study, participants need a method of filling out questionnaires repeatedly during the day. Usually, participants are signaled to do so at randomized time points, and thus also need a method to receive these notifications (sometimes also called bings, signals, or triggers).

Originally, ESM was performed with pen and paper supplemented with devices like pagers for signaling participants. The same can, however, be more efficiently achieved by a device that can combine the capabilities of signaling the participant, displaying questionnaires, and storing or relaying responses. Thus, recent studies often use smartphones running specialized applications for this purpose. Nonetheless, depending on how a smartphone is carried (e.g., on the body, in a bag) a participant might not notice a signal right away or the smartphone might be cumbersome to access. Specialized devices can support and complement ESM on smartphones e.g., [5,6]. Wearables, especially when worn on the wrist, are unobtrusive when not in use but always at hand when needed. With regard to the issues smartphones may face, wearables offer a lower burden for participants. 

A wearable in this context is a device that is somehow affixed to, or worn by, a participant. This includes smartwatches, which we define as a device worn on the wrist that has the capability to display information and have the user interact with it, alongside some computing power. They share the capabilities of regular watches (i.e., display time and other useful information), but are more ubiquitous by way of allowing the use of computing, to display and interact with arbitrary information (e.g., notifications from paired devices, relevant events) rendering them potentially useful for self-reports, such as those used in ESM designs. Smartwatches are, however, distinct from other devices with a similar form factor, such as screenless, one-button wearables, which can also be used for self-reports [7,8], or wearables with a clear sensor-based focus (e.g., pedometers, actigraphs in sleep research).

An early comparison of the ESM on multiple different devices found a trend of higher compliance on wearables like smartwatches or the Google Glass compared to smartphones [9]. Another result of this study was the significant difference in the number of received notifications between devices, with smartwatches receiving less notifications. Small batteries combined with improper charging routines resulted in more frequent loss of power in smartwatches, leading to scheduled notifications not being triggered.

One study also demonstrated good acceptance of ESM on smartwatches, even in a clinical sample. A sample of older adults reported to be largely satisfied with the use of a smartwatch for self-reports, despite potential issues like small screens [10].

A further route for employing wearables like smartwatches are microinteractions. These are defined as interactions by a user with a device that can be completed within four seconds, including retrieving the device from its storage (e.g., pocket, bag) [11]. For example, this can be implemented by using one-item questionnaires. The use of microinteractions could reduce participant burden, and smartwatches are a good candidate for implementing microinteractions, as they have minimal retrieval time. Indeed, when comparing ESM using microinteractions on smartwatches to classical multi-question ESM on smartphones, smartwatches do reach significantly higher compliance, even though the number of notifications is much higher due to their one-item questionnaire design [12]. In this study, microinteractions on smartwatches were realized by questionnaire items not being answered all at once at one specific time but being split up in single-item assessments over multiple time points. The higher compliance observed seems to be driven by the microinteractions, as the same benefits could not be observed when classical multi-question ESM was used on smartwatches [13]. Microinterations-based ESM on smartwatches has since successfully been used in a large-scale study over 12 months [14].

### 1.3. Programmable Smartwatches

Most commercially available smartwatches run on the Android Wear Operating System (OS). While applications can be developed for Android Wear-based smartwatches, these run on-top of an OS layer. However, so-called development devices (i.e., devices intended as a platform to test newly developed systems) allow the creation of custom firmware (usually within a software framework), thereby giving greater control over the device. Several programmable wearables are available in a smartwatch form factor. These options include commercially available devices, as well as projects requiring production and assembly of parts.

Two examples of the latter category are the Open-Smartwatch [15] and the ESP32 Smartwatch [16]. Both projects provide schematics, files to print circuit boards, and to 3D-print casings, as well as software. However, this do-it-yourself approach seemed too complex and detailed for our application of an easily available device.

Commercially available hardware include Bangle.js [17] (Espurino Shop, Culham, UK), the PineTime SmartWatch [18] (Pine Store, Hong Kong, China), Watchy [19] (SQFMI, New York, NY, USA), and the LilyGo T-Watch 2020 [20] (LilyGo, Shenzhen, China) to name just a few. All of these come with software resources to program the devices, mostly via developing low level firmware. The firmware presented in this paper is developed for the LilyGo T-Watch 2020, specifically Version 2. The T-Watch 2020 uses a ESP32 microcontroller (Espressif Systems, Shanghai, China) as its main processor. The T-Watch 2020 features a 1.54-inch (30.12 mm) 240 × 240 capacitive touchscreen for displaying information and receiving user input. The device can be programmed via a micro-USB port, which is also used for loading the battery. Beyond that the device possesses many other features common in smartwatches, e.g., an accelerometer sensor, bluetooth and Wi-Fi capability, and a vibration coin motor for haptic feedbacks.

However, one important factor for this choice is a micro Secure Digital memory card (i.e., microSD) slot featured by the device, a feature uncommon in smartwatches. This feature is the key in making the device autonomous, which reduces overhead (e.g., no need for servers and web interfaces) and prevents compatibility issues (e.g., paired smartphones losing connection to the smartwatch). Using a microSD card allows easy data transfer to and from the device. Furthermore, if used for scientific research, data security and anonymity are aspects often demanded by institutional review boards (IRB), which can be more easily guaranteed by using local storages of data such as SD-card memories. 

### 1.4. Other ESM Systems for Smartwatches

The fact that several studies have used the ESM on smartwatches or have developed software to do so shows, that the smartwatch form factor is of great interest in the context of self-reports. As mentioned above, early studies implemented basic ESM items ( two-dimensional grid and five-point Likert scale) on smartwatches [9], as well as items for microinteraction-based ESM (single items consisting of an item text and a list of answer options) [12,13,14]. Further design prototypes for response formats on smartwatches (e.g., different touch or motion-based inputs) were developed and tested in different hypothetical situations (i.e., walking, gaming, and social chatting) [21]. Beyond basic research some projects have tried to provide solutions for specific applications. Frameworks are available that combine ESM data and other real-time data, both collected by smartwatches, such as heart rate data [22] or mobility data from accelerometer sensors [23]. Another project features premade prompts (i.e., a fixed set of available items) for the assessment of participants’ comfort via the FitBit device [24]. A recent project also provides a general-purpose solution [25].

All except one of these projects used Android implementations for commercially available smartwatches. Only the FitBit-based Cozie project is available in an open-source repository [24], and others provide information that software is available upon request [23,25]. Contacting the remaining authors revealed that some of the other software would also be available upon request [12,13,22]. In summary, while several projects are available, there still is a lack of a tool combining the advantages of previous solutions, i.e., easily available, easy to use, easily extensible, user friendly, and a general-purpose solution for conducting ESM studies on smartwatches.

### 1.5. Free and Open-Source Software

There are two terms in the realm of software describing similar ideas. One such term is “Open Source.” This term mainly refers to the public availability of a software’s source code, thus allowing modifications to the code, redistribution, etc. [26]. The other term is “Free Software”, which requires similar freedoms of redistribution, modification (which requires the source code to be publicly available), as well as the freedom to use the software for any purpose [27].

Both “Open-Source Software” and “Free Software” overlap in their definition, leading to the term “Free and Open-Source Software” (often shortened to FOSS) to be widely adopted [28,29]. If a software is considered free and/or open-source depends on the license under which it is distributed. Both the Free Software Foundation and the Open Source Initiative maintain lists of compatible licenses (which largely overlap) [30,31].

The firmware presented here is licensed under the MIT License (see https://mit-license.org, accessed on 2 March 2022), which is considered a FOSS license. This allows this tool to be easily available for anyone interested in using it and even open for collaboration for anyone willing to improve it based on their own experience. Accessing, using, and improving upon other people’s work is a given in the scientific community, making it a natural choice to publish this project as FOSS. The project repository, along with instructions for using the project, is available athttps://github.com/KL-Psychological-Methodology/TWatch-2020-ESM (accessed on 2 March 2022).

### 1.6. Pilot Study

In order to compare the introduced T-Watch smartwatch to more established modes of data collection by using smartphones, we conducted a proof-of-concept study using a between-subject experiment. In this experiment, participants in both groups completed a week-long ESM study on their respective devices by answering questions about their affect (positive, negative mood) in a signal-based fashion, multiple times a day. They also answered questions about the ESM procedure itself (e.g., problems, participant burden) in an end-of-day questionnaire. We chose the construct of affect as it can be expected to vary throughout the day, thus justifying frequent measurements. Additionally, affect has also been used as a construct in previous studies for similar reasons [12,13].

Based on previous research we expected similar levels of compliance for smartwatches and smartphones [9,13]. Therefore, our main hypothesis was that there is no difference in compliance to signal-based questionnaires between a smartwatch group and a smartphone group. Further, we expect that the easy access to the smartwatch (compared to a smartphone) as a wearable will reduce the overall perceived burden. We therefore hypothesized that participants would report lower perceived burden in the smartwatch group than in the smartphone group at the end of each day. We also hypothesized that participants in the smartwatch group would report higher levels of burden in relation to maintaining the device than participants in the smartphone group and would further fail to keep their device charged more often (i.e., their device would lose power). Lastly, using smartwatches (instead of smartphones) should not impact the measurement process itself (e.g., higher negative reactance due to the haptic bings on smartwatches). Therefore, as an example, we assessed participants’ actual mood (i.e., positive and negative affect) several times a day. If smartwatches are a valid form of data collection, the measurement process should not affect the results. Our hypothesis was therefore that positive and negative affect would not differ between participants in the smartwatch and smartphone groups.

## 2. Materials and Methods

### 2.1. The Used Firmware

The following section gives a brief overview of our FOSS ESM firmware for the LilyGo T-Watch 2020 V2. See the Supplementary Material for a detailed overview.

We focused on several aspects in the development of this firmware. First, it should be user-oriented, and thus be usable with minimal or no programming skills. Second, it should be autonomous, meaning no additional hard- or software is necessary for operating the smartwatch. This autonomy also allows for a third goal, security, as no third parties are involved, and data is never transmitted over the internet. Third, it should provide researchers with common tools to implement ESM designs.

The general handling of the smartwatch for a study is relatively simple. After a one-time firmware upload the device can be configured for a specific study by creating a configuration file and storing it on the device’s SD-card. The device will read the configuration from this file and present notifications and corresponding questionnaires to the participant accordingly. The participant’s responses, as well as logs of these events, are stored on the same SD-card in CSV format, allowing easy access to the data after study completion.

The firmware is capable of handling event-based as well as signal- or interval-based schedules (via a list of available questionnaires or via fixed-time or pseudo-randomly timed notifications). Questionnaires can consist of multiple items that are displayed in a one-screen-one-item design. The firmware is capable of displaying items with four different response formats: Likert scale, visual analogue scale, number input, and text options (i.e., similar to a dropdown list). The firmware also handles a standby mode when the device is not in active use to conserve power, and wakes the device for elicited notifications, which alert the user via a haptic signal using the device’s coin vibration motor.

### 2.2. Participants

A total of 14 participants took part in this study (five male, eight female, one diverse, $M_{age}=35.1\;\mathrm{years},SD_{age}=11.7\;\mathrm{years}$). Due to the low number of subjects, we collected sociodemographics separately from the smartphone/smartwatch data in order to guarantee anonymity. One participant in the smartphone group experienced an error with the software, which prevented the device from triggering notifications after a short while. Therefore, data from this participant had to be excluded, resulting in a total *N* = 13 participants (seven in the smartwatch condition, six in the smartphone condition).

### 2.3. Design and Procedure

In the smartwatch group we handed out the system described above, i.e., T-Watch 2020 V2 with the described ESM firmware. In the smartphone group, participants used their own smartphones, using the application ESMira (Versions 2.4.2.3–2.4.3.0) to conduct the study. ESMira is a FOSS application developed for ESM studies, available both for Android and iOS [32].

In an introductory meeting, participants were randomly assigned to either the smartphone or the smartwatch condition. The assignment happened according to a balanced, randomly permuted list. In the smartphone group, participants were then assisted in setting up the ESMira application on their smartphone, as well joining the study in ESMira. In the smartwatch group, participants were handed the T-Watch, and instructed on its use. 

The behavior of the devices was set up to be as similar as possible, according to device limitations. The biggest difference was that the smartwatch used a one-screen-one-item design, while the smartphone presented all items on a single scrollable screen. Both devices would notify the participants in a signal-based fashion five times a day, pseudo-randomized within five consecutive two-hour time slots from 9:00 a.m. to 7:00 p.m. The notification remained for 15 min, or until answered or dismissed. Two reminders repeated the notification in intervals of five minutes. When interacted with, the notifications would lead to the questionnaire on both devices.

The interval-based end-of-day questionnaire, containing all questions regarding burden, was scheduled to trigger as a fixed-time notification at 7:00 p.m. This notification was active for 60 min, and a reminder repeated the notification after 30 min.

The whole study procedure lasted for seven full days.

### 2.4. Measures

#### 2.4.1. Positive and Negative Affect Schedule (PANAS; Signal-Based)

The international PANAS short form (I-PANAS-SF) [33], was used as a repeating questionnaire. The adjectives were used in the German translation provided by Krohne et al. [34]. The instruction was phrased so it would ask for state affect (“Please indicate how much you currently feel the following emotions.”). On smartphones, this instruction would appear as a header, after which the 10 adjectives were shown on a single page, each alongside a five-point Likert scale. On the smartwatch, the instruction was first shown as its own text prompt, followed by each individual adjective alongside a five-point Likert scale (one-screen-one-item design). The items were presented in a randomized order each time on both devices.

#### 2.4.2. Assessment of Burden (Interval-Based)

Participants filled out an end-of-day questionnaire about the current day, asking them the following questions:

1. Today I felt burdened by the smartwatch/my smartphone.
2. Today I felt that the notifications interrupted my everyday life.
3. I felt the number of notifications was …
4. The smartwatch/my smartphone was turned off since the last end-of-day questionnaire because the battery was empty.
5. It was easy to ensure that the smartwatch/my smartphone had enough power.

Questions 1, 2, and 5 were answered on a VAS, ranging from 0 = “not at all” to 100 = “very much”. Question 3 had the answering options “too much”, “appropriate”, and “too little”. Question 4 was a “yes/no” question (implemented as two-point Likert scale).

Questions 1–3 were intended to ask for general burden due to participation. Questions 4 and 5 were intended to assess how participants handled recharging their devices, and the burden caused thereby.

#### 2.4.3. Compliance Measures

Both the smartwatch firmware presented here, as well as the ESMira smartphone application create logs of triggered signals and reactions to them. These logs were used to assess the amount of received and answered notifications.

## 3. Results

### 3.1. Compliance and Completion

We first compared the number of triggered notifications to the number of scheduled notifications in each group. Each participant took part for seven days, with five scheduled notifications per day, for a total of 35 scheduled notifications per participant. Therefore, a total of 245 notifications were scheduled across all participants in the smartwatch condition (seven participants) and 210 were scheduled across all participants in the smartphone condition (six participants). Log files showed that 240 notifications were triggered in the smartwatch condition (98.0%) and 203 notifications were triggered in the smartphone condition (96.7%; see Table 1). A χ²-test showed that the two groups did not significantly differ in the ratio of received notifications to scheduled notifications, χ² = 0.32, *df* = 1, *p* = 0.573.

Compliance was assessed as the fraction of responses relative to the number of scheduled notifications. As the number of received notifications can be lower than the number of scheduled notifications, we also calculated completion as the rate of responses relative to the number of received notifications. See Table 1 for an overview.

We performed a χ²-test to test for a relationship between group and compliance, which indicated a significant group-dependence (χ² = 24.63, *df* = 1, *p* < 0.001). The risk ratio of that relation is *RR* = 1.39 (i.e., participants in the smartwatch group were 1.39 times more likely to answer a scheduled notification as participants in the smartphone group).

### 3.2. Perceived Burden

Several items concerning perceived burden were collected as part of an end-of-day questionnaire. This questionnaire was part of the used ESM protocol, and thus could also be missed by participants. Each participant could answer the end-of-day questionnaire up to seven times, once for each day they participated. This means a total of 49 scheduled questionnaires in the smartwatch group, of which 42 were answered (85.7% compliance). A total of 42 questionnaires were scheduled in the smartphone group, 30 of which were answered (71.4% compliance). Although the compliance was descriptively lower in the smartphone group, the difference was not statistically significant, χ² = 1.996, *p* = 0.158. The following analyses were based on collected responses, with missing values being omitted.

The first item asked for general burden (“Today I felt burdened by the smartwatch / the smartphone.”). A permutation test found no significant difference between the smartwatch group (*M* = 25.94, *SD* = 18.28) and the smartphone group (*M* = 33.60, *SD* = 21.17), *Z* = −1.548, *p* = 122.

The second item asked whether participants felt interrupted by the notifications (“Today I felt that the notifications interrupted my everyday life.”). Although participants in the smartwatch group descriptively reported more feelings of interruption than the smartphone group (smartwatch: *M* = 31.76, *SD* = 22.24, smartphone: *M* = 29.71, *SD* = 25.56), this difference was not statistically significant (permutation test: *Z* = 0.355, *p* = 0.723).

The third item was aimed at finding out whether the number of notifications was subjectively too high for participants (“The number of notifications was … too much/appropriate/too little”). Table 2 shows the distribution of answers. A Mann–Whitney *U*-test found no significant difference between the answers in the smartwatch group (*Mdn* = 2) and answers in the smartphone group (*Mdn* = 2), *U* = 617, *p* = 0.211. 

As we are looking at this in the context of burden specifically, we also dichotomized the answers by combining the answer options for an “appropriate” number of notifications and “too little” notifications. Thus, we calculated a Fisher’s exact test to assess whether one group more strongly felt that the number of notifications was too high. However, this test was not significant, indicating no difference in this regard between groups, *p* > 0.999.

The fourth item asked whether the device had lost power (i.e., has completely discharged) within the last day (“The smartwatch/my smartphone was turned off since the last end-of-day questionnaire because the battery was empty.”). This happened five times in the smartwatch group (out of 42 answered questionnaires), and only one time in the smartphone group (out of 30 answered questionnaires). A Fisher’s exact test shows no significant difference between groups, *p* = 0.228.

The fifth and last question was concerned with the subjective experience of burden from having to charge the devices (“It was easy to ensure that the smartwatch / my smartphone had enough power.”). A permutation test found no significant difference in burden from recharging the device between the smartwatch group (*M* = 64.45, *SD* = 33.19) and the smartphone group (*M* = 70.70, *SD* = 33.74), *Z* = −0.776, *p* = 0.438.

### 3.3. Positive and Negative Affect

Lastly, we analyzed participant’s responses to the signal-based PANAS questionnaires. We used random-intercept multi-level linear regression models, with individual measurements of positive or negative affect, respectively, (level 1) nested within participants (level 2). Device type (smartphone vs. smartwatch) was included as a predictor variable. The analyzed data are based on full sets of five items of each scale, i.e., if one of the items to calculate positive or negative affect was missing, that measurement was discarded for the respective scale.

According to the multi-level linear model, the groups did not differ significantly in either positive or negative affect (Table 3).

## 4. Discussion

In this paper we presented a FOSS, general-purpose ESM firmware for a commercially available programmable smartwatch. This provides researchers from different disciplines with the possibility of realizing smartwatch-based in situ data collection without detailed programming skills, i.e., one of this firmware’s main goals was to provide researchers both flexibility and autonomy in the realization of their empirical research studies.

Usability for participants is an important factor, as the use of the smartwatches should not negatively impact a study. We hypothesized that for appropriate study protocols using only a few and short questions, the ease of access should provide a benefit over the general ease of use for inputs on alternatives like smartphones. While we predicted that there would be no difference in compliance between the smartwatch and smartphone groups, based on previous research [9,13], the use of smartwatches resulted in significantly better compliance than on smartphones in our pilot study.

Apart from compliance, we also investigated subjective participant burden. While we hypothesized that smartwatches would lead to lower general burden and feelings of interruption, but higher feelings of burden due to the need for maintenance, we found no significant differences in our measures of subjectively experienced burden or the number of reported charging failures. We also found no significant differences between groups in positive and negative affect. This is in line with our hypothesis that the device type should not influence immediate response behavior. Beyond results that suggest a benefit of using the presented smartwatch solution in appropriate situations, our firmware approach has the following benefits.

### 4.1. Benefits of this Firmware

#### 4.1.1. Ease of Use

The main goal of the presented smartwatch firmware development was to make these smartwatches easily usable for empirical studies in general, and ESM design in particular, without the need for detailed programming skills. There are some technical steps involved in uploading the firmware to the device, but this is a one-time step (apart from occasional firmware updates). After its initial setup the device can be configured using configuration files as detailed in the sections above. A configuration application should also make this as easy and intuitive as possible.

#### 4.1.2. Autonomy

The presented firmware is designed to work autonomously. Most regular smartwatches work in tandem with a paired smartphone. This can cause issues, as seen for example in Ponnada et al. [13], where unforeseen incompatibilities between the smartwatches used and certain smartphone models caused connectivity issues and thus caused drop-outs. The necessity of pairing may further restrict the participant pool, as only participants with certain smartphone brands, that are willing to install the required applications on their phones, can participate.

Driving the smartwatch via a paired smartphone has its benefits, like the ability to run more complex and powerful software, as well as the ability to upload data almost in real-time, allowing for online monitoring of ongoing data collection. However, this system also has drawbacks. Two devices required to work together introduces additional points of failure, like software incompatibilities, issues of unavailable connections, and so forth. The necessity for a constant wireless connection also increases the power demand of the wearable, thus further shortening its battery life. Acquiring the data may also get more complicated, as data sent via the internet necessitates a webserver compatible with the software used on the smartphone or smartwatch.

In contrast, the T-Watch 2020 V2 has an internal slot for a microSD card, which the firmware uses to retrieve configuration and localization files, and to save generated data. Configuration and localization files are in JSON format, meaning they can theoretically be created and edited with any text editor. The generated data is saved in CSV format and should therefore be compatible with most statistical software. The use of a microSD card makes it easy to transfer these files between the device and a researcher’s computer. The reduction of dependencies allows researchers to operate the device without any specific additional hardware or software.

This makes the used system an autonomous device, which can be handed to the participants as-is (optionally with the addition of a micro-USB cable for charging). There is no need for setup or pairing, reducing the burden of maintenance for the participants to charging the device and remembering to wear it.

Apart from reducing points of failure, this also increases data security. Data are only gathered locally and only transferred directly from the SD card to the computer. Therefore, they are never transmitted over the internet and cannot be accessed by third parties.

#### 4.1.3. Extensibility

The provided firmware is FOSS software, and as such it is modifiable and extensible. This ranges from small modifications to larger additions of functionality. For example, some string lengths or arrays are statically allocated, resulting in fixed maxima. Most of these maxima are defined in a single file, making it easy to alter them, should a specific use case require different maxima (i.e., a larger number of items per questionnaire).

Part of this modifiability is also the ability to change or extend the used font. The default firmware should be able to display most Latin-based scripts. If any special fonts or glyphs become necessary users can include them in their custom firmware versions. 

Furthermore, the project’s open-source nature ensures its longevity, as it can be maintained or forked by anyone willing and able to further develop this project.

### 4.2. Limitations

The presented study has several limitations. One is the small sample size of the pilot study. This limitation also affected analyses where non-parametric tests with comparatively less power than parametric tests had to be used. A larger sample size might have allowed the use of multilevel modeling, as was used with PANAS data.

Another limitation was the study time. While a week is not uncommon for ESM studies [35], a comparable study by Ponnada et al. [13] used a study duration of four weeks. In contrast to the present study, Ponnada et al. found no difference in compliance between ESM on smartwatches and smartphones. However, the authors did mention that they observed higher initial compliance in the smartwatch group, but this compliance dropped quickly over the course of their study, overall averaging to similar levels as smartphones. It is therefore possible that the effect in compliance observed in this study could be an initial effect, while the overall effect would be alleviated in longer study durations.

It should also be mentioned that this study was conducted during the COVID-19 pandemic. While there was no active lockdown at the time the data was collected, the possibility of altered participant behavior as a consequence cannot be discounted. For example, prolonged time at home due to reduced social contact and working from home could lead to some participants not carrying their smartphones close by, which could reduce compliance due to missed notifications.

Apart from study-specific limitations the smartwatch-approach itself has some limitations too, compared to smartphones. One of these is the smartwatch’s generally small form factor. The necessity for small size leads to smaller batteries and screens compared to smartphones. According to our experience with the described devices and use-case, the smartwatches need to be recharged daily to remain operational, despite their lower power consumption.

The smartwatches’ screens are substantially smaller than modern smartphone screens in both size and resolution, which presents another constraint. It limits the amount of information that can be displayed at once, resulting in a need to adapt item texts and response options, which may not always be possible. If images are displayed on such a small screen, they lose a lot of detail and may not be properly recognizable. Apart from challenges with output, the small touchscreen as the sole primary input presents additional challenges, such as elements of the graphical user interface needing to be small due to the limited space available, while users use their fingers for inputs, thereby covering a proportionally large part of the screen. This is noticeable in items like the visual analogue scale or the Likert scale. Precise inputs are further impeded by devices differing in factory touchscreen calibration. The small area amplifies the effect of slight offsets in calibration between the capacitive touchpad and display. Indeed, some participants reported problems in consistently selecting the outer ends of scales on specific devices.

These device limitations combine to reduce the ease of use in interacting with the smartwatch. While a smartwatch, as a wearable, may benefit from its unobtrusiveness in short interactions, long interactions, e.g., long questionnaires, can feel tedious. Therefore, not only are the items limited in their content, i.e., short instruction and response texts, but the viable number of items in a questionnaire is limited on smartwatches as well.

Lastly, there are limitations of the approach of using the presented firmware solution specifically. The autonomous offline approach has its own drawbacks. The possibility to monitor incoming data during ongoing studies would for example enable researchers to detect improper configurations early and update them remotely, increasing the amount of usable data collected. Further, executing configuration-related code centralized on a more powerful computing device can also allow for more complex study designs, e.g., adaptive designs. However, the autonomous offline approach prevents the use of these options.

Furthermore, the firmware has a specific device as a dependency. We chose the T-Watch 2020 V2 for its features, especially the microSD card slot, hence we built the firmware specifically for this device. While this allows greater control, and adjustment, this also reduces the risk of the firmware becoming obsolete together with the device. The device will likely be discontinued at some point or can otherwise become hard to acquire. This device-dependence is somewhat mitigated by the fact that the firmware is based on the ESP32 microcontroller architecture, and in large part is not device-dependent. Therefore, it could likely be ported to a similar platform using the same chip architecture.

### 4.3. Future Outlook

The current firmware is a general-purpose tool to conduct a range of common ESM designs. However, the T-Watch 2020 V2 offers capabilities currently not used by the firmware, which might be utilized in future development. One such feature is the accelerometer. Accelerometers are very commonly used for passive online monitoring of movement. A combination of active self-reports and passive acceleration data is thus a possibility.

Another use of the accelerometer would be the implementation of a Physical Analogue Scale (PAS) item, as introduced by Stieger et al. [8]. Using the physical arm angle for an analogue scale, the PAS could compensate for the small screen by allowing a physically bigger space for answer options. As mentioned above, the T-Watch 2020 V2 also has a GPS receiver, allowing the device to measure its location. Utilizing this GPS system could allow relating answered questionnaires to location data, or to track the general movement of participants.

Besides the utilization of technical capabilities of the device, the firmware might also be expanded by different modes of operation. The most important example would be support for microinteraction-based ESM. While we chose to focus on a general-purpose solution first, in order to cover a wider range of applications, the advantages of microinteraction-based ESM over classical ESM on smartwatches make it an attractive next step. To function for proper single-item microinteractions, such an option would skip the display of notifications in favor of directly displaying an item, thereby reducing the need for an additional interaction. This could further improve compliance, even compared to single-item questionnaires preceded by a notification, as single-interaction settings without screen changes are known to reduce perceived burden [36].

### 4.4. Summary

Overall, the presented project has multiple benefits. It introduces a general-purpose device including our open-source framework, which allows a multitude of ESM designs to be realized. The setup process is user-friendly, and configuration is made easy by a configuration application. Configuration can also include language localization, allowing the system to be easily used in other languages. After setup, the device runs autonomously, meaning there is no risk of compatibility issues, and that data are secure. As shown in our proof-of-concept study, for short questionnaires the system is on par with smartphones in terms of compliance. While this system’s applicability is dependent on a suitable design (e.g., short questionnaires with simple items), it should provide a good complementary approach to smartphones in these situations. We therefore hope that others will adopt this firmware for their empirical research and find it a useful extension to the methodological repertoire of data assessment tools.

## Figures and Tables

**Table 1:** Number of scheduled, received, and answered notifications, alongside resulting compliance and completion.

Group	Scheduled (S)	Received (R)	Answered (A)	Compliance (A/S)	Completion (A/R)
Smartwatch	245 (100.0%)	240 (98.0%)	193	78.8%	80.4%
Smartphone	210 (100.0%)	203 (96.7%)	119	56.7%	58.6%

**Table 2:** Distribution of answers regarding the appropriateness of the number of notifications.

Group	“Too Much”	“Appropriate”	“Too Little”
Smartwatch	3	35	1
Smartphone	2	24	4

**Table 3:** Results of multi-level models of positive and negative affect.

	Fixed					Random
	*B*	*CI*	*SE*	*t*	*p*	*SD*
Positive Affect						
Intercept	17.82	15.53–20.12	1.16	15.31	<0.001	2.74
Device type	−1.68	−5.16–1.79	1.58	−1.07	0.309	
Negative Affect						
Intercept	8.52	6.91–10.13	0.82	10.43	<0.001	1.89
Device type	−1.73	−4.16–0.70	1.11	−1.57	0.146	

Note. Device type: Smartwatches are coded as 1, smartphones as 0.

## Data Availability

The data presented in this study are openly available in OSF at https://osf.io/n6qfk/ (accessed on 2 March 2022).

## Supplementary Materials

The following supporting information can be downloaded at: https://www.mdpi.com/article/10.3390/s22051980/s1, Figure S1: Excerpt of configuration file and item displayed on the screen; Figure S2: Configuration application; Figure S3: Pictures of the device showing the main screen; Figure S4: Scale items (5-point Likert scale and VAS); Figure S5: Numeric input and options input items; Figure S6: Device displaying a notification. Code and documentation are available in the public GitHub repository: https://github.com/KL-Psychological-Methodology/TWatch-2020-ESM (accessed on 2 March 2022).
