# Supplementary material for: Self-Reports in the Field Using Smartwatches: An Open-Source Firmware Solution
Source: Sensors (Basel). 2022 Mar 3;22(5):1980. doi: 10.3390/s22051980 (PMC8915061; doi:10.3390/s22051980)
Supplement: Supplementary file 1 [file sensors-22-01980-s001.zip › sensors-1546639-supplementary.pdf]

Supplementary Materials

Firmware Overview

The following section details the capabilities, features, and behaviors of our FOSS ESM firmware for the LilyGo T-Watch 2020 V2. The firmware utilizes LilyGo's official software library for the T-Watch. This library optionally features the Light and Versatile Graphics Library (LVGL, https://lvgl.io), which the firmware uses for handling all its graphical user interfaces.

Firmware upload

To use the firmware on the T-Watch 2020 it first needs to be flashed, i.e., uploaded to the device from a computer. There are several ways to achieve this. The easiest and most user-friendly way is to use Espressif's Flash Download Tool, available on their website. This tool for Windows allows to upload the compiled firmware binary to the Device directly. The necessary steps and settings for this are detailed in our project's GitHub repository.

The second way is to use the PlatformIO plugin, available for the freely available software package Visual Studio (VS) Code which, in combination with PlatformIO, is usually used to program and flash devices such as ESP32 microcontrollers. The repository contains a PlatformIO project that can easily be compiled and uploaded to the device. Instructions for this process are available in the project repository as well.

Configuration file

Configuration of the device happens via a configuration file stored on the microSD card, which the device will automatically try to load if one is inserted. This configuration file is a plain text file written in JSON (JavaScript Object Notation) format. This file can either be created via a simple text editor or by using a provided questionnaire-programming tool (see the following section 1.7.3.) This provides a good balance between being human readable and easy to parse by the device.

The configuration file is structured in a nested fashion. The root object will contain the study name, and configuration options for the whole device. An array of questionnaire objects is nested in this root study object. They configure the names of each questionnaire, whether it should be listed as an event-based questionnaire, and if it is active or not. Inactive questionnaires are simply ignored by the software, allowing the option to leave often-used questionnaires in a configuration file, so they do not have to be re-entered if the same configuration file is adapted for a new study at a later date.

Questionnaire objects also contain two arrays, one for the definitions of notifications, and one for the items contained in the questionnaire. The object in the alarms array defines when notifications for the respective questionnaire should be elicited, and how these should behave. The item array contains objects with the information necessary to build the items on-screen. Figure 1 shows an example of a questionnaire definition from a configuration file, alongside the corresponding item.

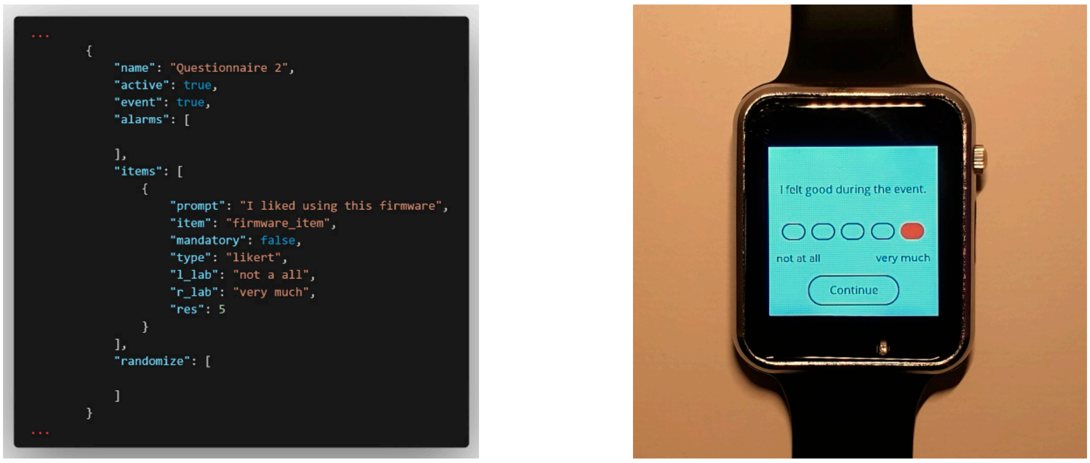

(a) (b)

Figure S1. (a) excerpt of configuration file; (b) item displayed on the screen

Configuration tool

Configuration files are text files that allow users to create and edit them in any text editor. However, to simplify this process we developed a FOSS tool that allows the user to create and edit configuration files, making it easy to create the files in accordance with the format the device expects. This tool offers a graphical user interface in which the configuration of questionnaires, items, and so forth, can be set in a point-and-click method.

This tool was built in the Godot Game Engine (https://godotengine.org), a FOSS application usually used for game development. However, Godot is also suited for application development, and its export options allow deployment of our configuration tool on all major Operating Systems. Figure 2 shows an example of the tool's workflow.

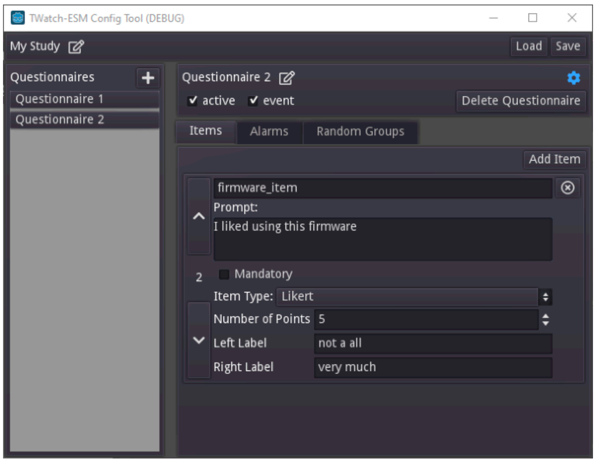

Figure S2. Configuration Application

Language Localization

Besides the configuration file, which is concerned with information about the behavior of the device, study administrators can place a language localization file (i.e., translation of default strings like "continue") on the microSD card. The firmware's default is English, and it defines all strings that are not study-specific, such as the names of options in the settings menu. Administrators can override these defaults via the localization file, which is also in JSON

format. As these values are unlikely to change from study to study, the localization file is separate to make reusing it easier.

Main Screen

During regular operation, the device will remain in standby mode most of the time, i.e., display and touch input are deactivated. When a user activates the Smartwatch via the power button, the device will turn on the display and show the main menu (unless the device should display a notification, see section 1.7.7.). This menu most prominently will feature a list of event-based questionnaires, if applicable to the study. This allows participants to select the appropriate questionnaire quickly when an event has occurred. See Figure 3 for images of the two main screen alternatives.

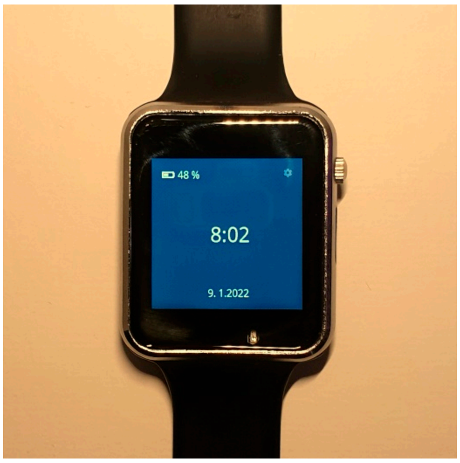

(a)

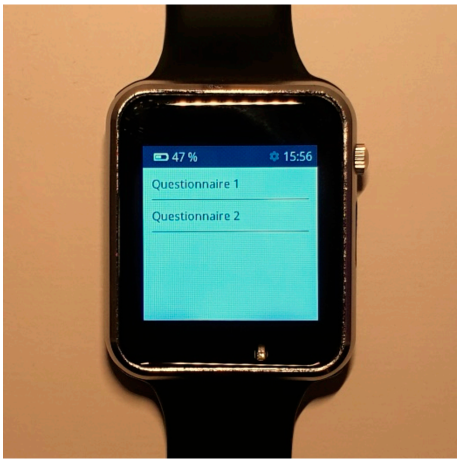

(b)

Figure S3. Pictures of the device showing the main screen: (a) for purely signal-based studies; (b) with event-based questionnaires available

Questionnaire and Item Types

Once a questionnaire is selected by tapping on the respective text on the touchscreen or accepting a notification, it will automatically load all items from the configuration file and display them sequentially. Each item displayed consists of an item text, a way to interact with the item (e.g., Likert-type answering scale), and a *continue* button. The *continue* button will display the next item(s) of the questionnaire (i.e., one-screen-one-item design). If the *continue* button is pressed on the last item, all questionnaire data will be saved to a data log file on the microSD card and advance to a screen telling the user that they have completed the questionnaire.

The firmware currently features four different item types: a Visual Analogue Scale (VAS), a Likert-type scale, an item type for numeric inputs, and an item type to select from different options (i.e., similar to a drop-down menu in HTML). In addition, there is a pseudo-item type for displaying a text message, with the intended purpose of supplementing other item texts (e.g., instruction-based text).

All items have an individual name for logging purposes together with an item text. See Figure 4 for pictures of the two scale item types (VASs and Likert scale).VAS items have a fixed range between 0 and 100, while the number of points on a Likert scale item can be set in the item's definition.

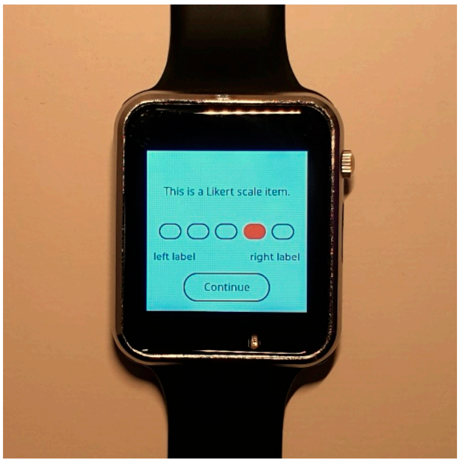

(a)

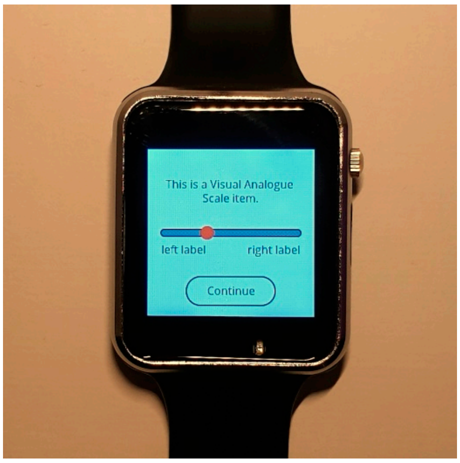

(b)

Figure S4. Scale items, which both allow to define labels for the scale ends: (a) a 5-point Likert-type scale item; (b) a VAS item

The numeric item allows users to input specific numbers, while the options item defines a range of selectable text options, similar to a drop-down menu. See Figure 5 for pictures of the both these item types.

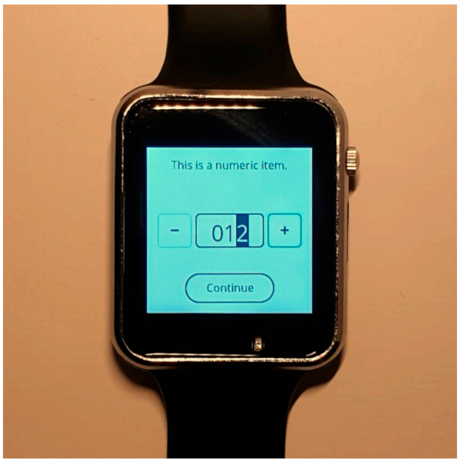

(a)

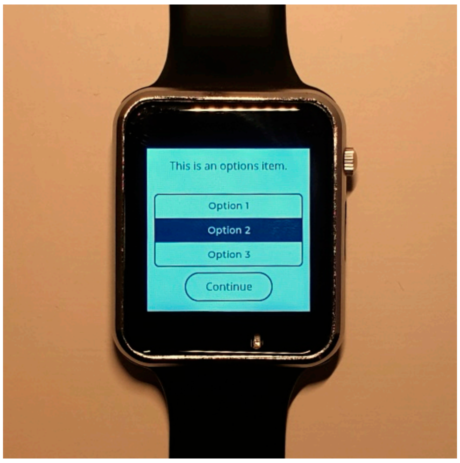

(b)

Figure S5. (a) The numeric input item allows users to select and manipulate individual digits on a presented number display; (b) The options item presents multiple text options in a roller, which users can select by swiping or tapping.

Notifications

Besides the handling of questionnaires, a key feature for performing ESM studies on the device is notifications (often also called bings or signals). The device will generate all notification trigger times for the following day at midnight during regular operation. The same trigger generation will also run at startup, meaning the system is robust against loss of power. A trigger in this context refers to the pre-generated, internally stored time a certain notification should be elicited.

Notifications alert the user by a haptic signal via the device’s vibration motor, and show a notification screen, as shown in Figure 6.

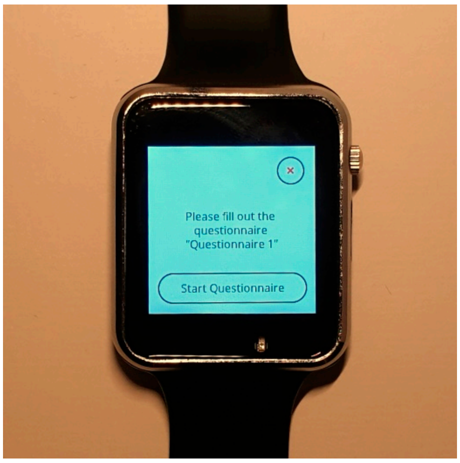

Figure S6. Device displaying a notification. Users can accept the notification using the “Start Questionnaire” button or decline it using the X-button.

When the device enters its standby mode by deactivating the screen, a wake-up timer will be set for the next notification trigger time. It will then wake up and notify the user via the vibration motor.

In the configuration file, each questionnaire can be defined with multiple notifications to be elicited. Each (fixed-time) notification is defined by a trigger time (in hours and minutes). This definition can be extended by a duration (in minutes) for pseudo-random timing. In this case, the notification will trigger a random number of minutes after the defined trigger time, up to the duration (i.e., the trigger time acts as the start time for a time slot of the set duration in which the notification can occur).

For each notification, an expiry time can be defined. Expiry time in minutes is the amount of time after the initial trigger, for which an unresolved notification will still be shown when the user wakes the Smartwatch up. This will be the case if the Smartwatch is sent to sleep because the user did not react to the notification or sent it to sleep themselves via the power button.

The notification can furthermore be extended by a number of reminders. In case the participant did not respond to a notification within the expiry time, the notification will be elicited again. This can happen multiple times, as often as it is defined in the notification’s configuration (in intervals defined by the expiry time). For example, if a signal is defined to expire after five minutes and to repeat twice, the notification will be triggered up to three times and available for up to 15 minutes total.

In order to evaluate response rates, the device logs every elicited notification in a log file. It will enter the initial notifications and reminders separately, as well as user reactions, i.e., accepting or declining a notification.

Further Firmware Features

Power and Sleep Mode

Due to the small form factor of the Smartwatch compared with Smartphones, the battery size and capacity are also reduced (in the present case 380 mAh). Therefore, the device's power consumption is a relevant factor. The firmware takes several steps to reduce power consumption to a minimum.

As the current version of the firmware does not use any radio communication, Bluetooth and WiFi are switched-off by default. Further, the device will enter sleep-mode either manually, when a user short presses the power button, or automatically after it has not detected any touch inputs for a set amount of time. While in sleep mode, the device will wake up either after the user presses its power button, or when a notification is triggered.

The ESP32 offers several sleep modes, with its deep-sleep mode substantially reducing power consumption. According to the ESP32's data sheet regular power consumption is between 20—68 mA, depending on clock speed (provided radio communications are deactivated; https://www.espressif.com/sites/default/files/documentation/esp32_datasheet_en.pdf). Light sleep should reduce this to 0.8 mA. Deep-sleep can further lower power consumption between 10—150 μA.

This makes the ESP32's deep-sleep mode very attractive for applications with a lot of inactive time, as it is the case in this project. However, using the deep-sleep mode would necessitate re-initializing the watch after wake-up. Due to the way the used library initializes the peripherals, this takes several seconds, introducing a delay. While this delay might not be noticeable when the device is woken by a notification, for user-driven (i.e., event-based) entries, the user would have to wait while the device seems unresponsive. Therefore, our firmware uses the ESP32's light-sleep mode. From our initial experience this is sufficient to keep the device powered for slightly more than a day, allowing continuous operation provided it is recharged daily.

Available font scripts

The software supports UTF-8, meaning the use of non-ASCII letters should be no problem. The only limitation is the glyph availability within the font. The font included in the source code was configured to include Unicode ranges Basic Latin, Latin-1 Supplement, Latin Extended-A, and Latin Extended-B. This should cover most languages with Latin-based scripts. The font files were created using LVGL's online font converter (a web-tool to create font files compatible with the graphics library). If other ranges become necessary, new font files including these ranges can be created using this converter and complied with into the firmware.

Adaptions if Version 1 of the T-Watch is used

We specifically chose Version 2 of the LilyGo T-Watch 2020 as target for this firmware, as the built-in microSD card slot makes handling the device very easy. Once the firmware is loaded on to the device, transfer of data to and from the device happens via files on the microSD card, eliminating the necessity for any special software on other devices to interface with the watch. However, in some cases researchers might prefer to either use Version 1 (because it is cheaper, or because it is simply what is available), or use Version 2 without an SD-card (as

was necessary in the case of one of our test devices, which would not recognize the SD-cards). In these cases, the device can utilize its internal flash memory via the SPIFFS library. This library allows storing data on the device itself.

After establishing a serial connection to the device, options in the device's settings menu can trigger the device to print the complete log file via the serial monitor, from where the data needs to be copied into a file on the PC. Unfortunately, this file system cannot easily be accessed by other devices, like is the case with the microSD card. Researchers need to upload the configuration files to the device via tools like the Arduino IDE or PlatformIO, similar to how the firmware itself can be uploaded. Similar tools need to be used to retrieve the logged data from the device.

While this process is more complex, it still extends the range of usable devices. Future development might yield companion Smartphone apps, which could simplify the process, and communicate with the device via Bluetooth.
